# *Erwinia mallotivora* sp., a New Pathogen of Papaya (*Carica papaya*) in Peninsular Malaysia

**DOI:** 10.3390/ijms12010039

**Published:** 2010-12-24

**Authors:** Noriha Mat Amin, Hamidun Bunawan, Rohaiza Ahmad Redzuan, Indu Bala S. Jaganath

**Affiliations:** Biotechnology Research Centre, Malaysian Agricultural Research and Development Institute, P.O Box 12301, General Post Office, 50774 Kuala Lumpur, Malaysia; E-Mails: hamidunb@yahoo.com (H.B.); rohaiza@mardi.gov.my (R.A.R.); indu@mardi.gov.my (I.B.S.J.)

**Keywords:** Carica papaya, Erwinia mallotivora, papaya dieback

## Abstract

*Erwinia mallotivora* was isolated from papaya infected with dieback disease showing the typical symptoms of greasy, water-soaked lesions and spots on leaves. Phylogenetic analysis of 16S rRNA gene sequences showed that the strain belonged to the genus *Erwinia* and was united in a monophyletic group with *E. mallotivora* DSM 4565 (AJ233414). Earlier studies had indicated that the causal agent for this disease was *E. papayae*. However, our current studies, through Koch’s postulate, have confirmed that papaya dieback disease is caused by *E. mallotivora*. To our knowledge, this is the first new discovery of *E. mallotivora* as a causal agent of papaya dieback disease in Peninsular Malaysia. Previous reports have suggested that *E. mallotivora* causes leaf spot in *Mallotus japonicus.* However, this research confirms it also to be pathogenic to *Carica papaya*.

## 1. Introduction

The genus *Erwinia* is classified in the family of *Enterobactericeae*, typically facultative anaerobic, gram negative rods possessing peritrichious flagella. Most members of this genus are naturally plant-pathogenic and plant-associated bacteria. *Erwinia* sp. are responsible for such basic types of plant disease as necrosis, blight, soft rot and wilt in a number of crop plants. For example, fire blight caused by *E. amylovora* is the most destructive disease in pear and apple orchards in many parts of world [1]. *E. chrysanthemi* pv. *zeae* and *E. corotovora* subsp. *carotovora* cause a highly destructive disease in maize and soft rot of potatoes as well as a wide range of fruits and vegetables [2].

Papaya (*Carica papaya*) is an economically important fruit crop grown in Malaysia with an export value of about RM100–120 million per year [3]. Currently, one of the major threats of this industry in Malaysia is papaya dieback disease. The common symptoms observed include greasy, water soaked lesions and spots on leaves, as well as foliar and angular lesions. These lesions can lead to secondary infection, which can eventually cause the death of the papaya plant. This disease has been a problem to papaya growers for almost a decade, destroying more than one million plants.

The disease was first identified in Malaysia near Batu Pahat, Johor in late 2003. Another incidence was later reported in Bidor, Perak, in October 2004. More recently, Maktar *et al.* [4] reported *E. papayae* as causing papaya dieback in Malaysia. However, they performed no significant biochemical tests to distinguish *E. papayae* and *E. mallotivora*, which are closely related species. They relied on two basic biochemical tests (oxidase and catalase) and the sequence similarity of the 16S rRNA gene to confirm *E. papayae* as a causal agent of papaya dieback in Malaysia. *E. papayae* was first reported by Gardan *et al.* [5] as the causal organism of papaya bacterial canker in the Caribbean region. However, the advanced stage of papaya dieback reported by Maktar *et al.* [4] had no canker symptom. Thereafter, no additional information on further occurrence of the disease has been reported until our finding, reported here, of *E. mallotivora* as a causal organism for this disease.

The aim of this study was, firstly, to isolate and identify the papaya dieback pathogen, and subsequently, to confirm its pathogenicity through Koch’s postulate. By combining phenotypic and genotypic information, we confirmed *E. mallotivora* as the causal agent of papaya dieback disease in Peninsular Malaysia.

## 2. Results and Discussion

**Figure 1:**
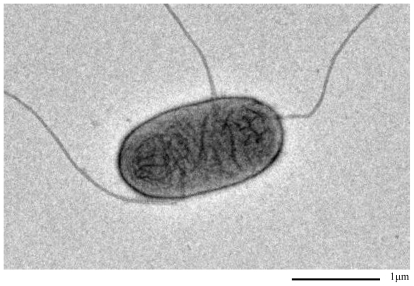
Transmission electron microscope image of the strain of *E. mallotivora* isolated from dieback-infected papaya tree.

Gram-negative and rod-shaped bacteria with a peritrichous flagella arrangement were consistently isolated from water-soaked lesions (Figure 1). The isolate from infected tissues produced hyaline colonies on Luria Bertani (LB) agar, typically with slow growth (2–3 days incubation). Colonies were creamy to white in color on King’s B [6] agar after 2–3 days incubation and on YBGA (0.7% yeast extract, 0.7% bactopeptone, 0.7% glucose and 1.5% agar, pH 7.2) medium after 3–4 days incubation at 25 °C. No water-soluble or non-water-soluble pigments were produced on King’s B medium. Biochemical analysis revealed that the strains are catalase positive and oxidase negative with the ability to form reducing compounds from sucrose. In addition, the strains produced acid from d-mannitol and utilized both lactate isomers, d- and l-lactate, which is consistent with the results reported by Goto [7] and Gardan *et al*. [5] for *E. mallotivora*. In contrast, *E. papayae* was unable to produce acid from mannitol, dl-lactate and sucrose-reducing compounds [5]. The apparent phenotypic characteristics of *E. papayae* and *E. mallotivora* are summarized in Table 1.

**Figure 2:**
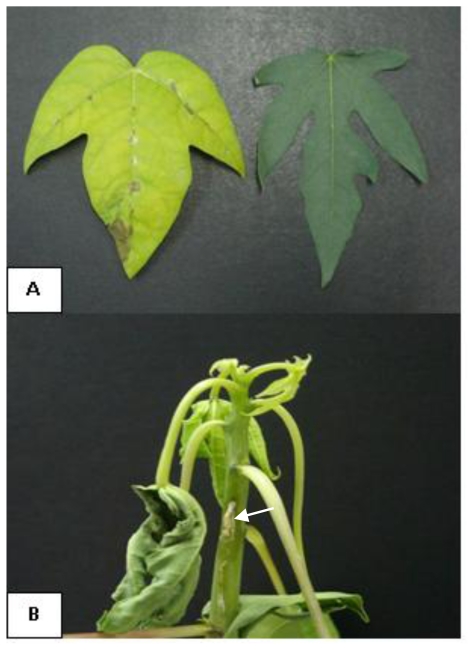
Papaya dieback symptoms caused by *E. mallotivora* (**A**) Leaf spots formed along the main vein of infected leaf (left) compared to a healthy leaf (negative control: right); (**B**) Greasy and water-soaked lesions leading to the destruction of papaya tree (Arrow).

Pathogenicity testing showed that the injected isolates formed brown spots on the leaves, water soaked lesions and greasy spots on the stem after 4–5 days of inoculation around the sites of inoculation and death of the plant occurred about 15 days after inoculation (Figure 2A, 2B). These symptoms observed are typical of those found in natural papaya dieback infection. Control plants showed no symptoms of the disease. *E. mallotivora* was recovered from the lesions as pure cultures, supporting our preliminary results that suggest that this bacterium is responsible for papaya dieback disease; the same pathogen has been reported to infect *M. japonicus* with only mild symptoms exhibiting as small lesions on the stem but not causing dieback of the infected shoot [7]. However, with papaya, *E. mallotivora* showed severe symptoms and caused dieback of the infected shoot, leading to the destruction of the plants without evidence of canker symptom.

*Erwinia* sp. have been long known as plant pathogens that produce a wide range of enzymes able to degrade plant cell wall components [8,9]. Leu *et al*. [10] reported that *Erwinia cypripedii* caused black rot on seedlings, trees and fruits of papaya in Taiwan. In 1982, Trujillo and Schroth reported two diseases. *Erwinia* decline of papaya (D strains) and the *Erwinia* mushy canker disease of papaya (MC strains), in Hawaii [11]. Webb [12] reported a species of *Erwinia* that caused angular water-soaked lesions on leaves and firm water-soaked cankers on the stems of papaya. Recently, the causal agent of bacterial canker in papaya was identified as a new bacterium species named *E. papayae* [5]. In this 1μm study, we report for the first time *E. mallotivora* as a new pathogen of papaya. The disease is transmitted from plant to plant or to other parts of the same plants, primarily by insects, birds, humans and rain splash [4,13]. Bacteria may enter through stomata, hydathodes, and lenticals and through wounds made by, for example, insects and hailstorms. From the leaf, the bacteria pass into the petiole and the stem, first colonizing and then moving through vessels of the plant.

**Figure 3:**
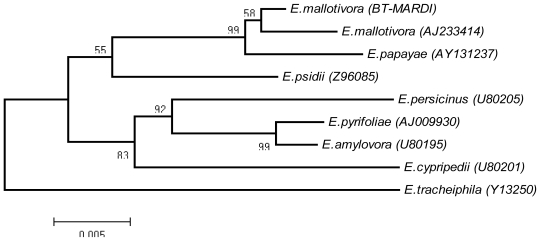
Phylogenetic tree based on neighbor-joining phylogram.

A BLAST analysis of the 1.5-kb 16S rRNA gene sequence showed high nucleotide identity (99%) to the 16S rRNA gene of *E. mallotivora* DSM 4565 (accession number AJ233414) and *E. papayae* (accession number AY131237). Phylogenetic analysis revealed that the *E. mallotivora* (BT-MARDI) from Malaysia (accession number HQ456230) belongs to the genus *Erwinia* and is united in a monophyletic group with *E. mallotivora* DSM 4565 and *E. papayae*. It showed that *E. mallotivora* and *E. papayae* are closely related species [5]. The group was supported with a high number of bootstraps in a neighbor-joining (NJ) tree (Figure 3). *E. mallotivora* (BT-MARDI) formed a robust cluster with the strain *E. mallotivora* (AJ233414).

## 3. Experimental Section

### 3.1. Bacterial Isolation

The strain was isolated from water-soaked lesions formed on the leaves, stems and fruits of naturally infected papaya plants at MARDI Research Station Serdang, Selangor, in early August 2010. Isolation procedures followed those of Agrios [14].

### 3.2. Koch’s Postulate

For pathogenicity testing, three month-old seedlings of *C. papaya* (cv. Sekaki) were infected with the bacterial suspension to determine the causative agent. Leaves of experimental plants were inoculated by pricking their abaxial surface with a sterile needle and injection with 50 μL of the inoculate suspension at a concentration of 1 × 108 CFU per mL into the apex of the seedlings, according to standard techniques described by [15]. Control plants were similarly inoculated with sterile water. Plants were incubated under greenhouse conditions at temperatures between 28 °C and 32 °C in the day and 25 °C and 28 °C at night. Using the same technique, bacteria were reisolated once symptoms appeared. Selected biochemical study of this bacteria was performed according to Gardan *et al*. [5,16] and the bacterial strain was identified using the API 20E system (BioMérieux, USA). Negative staining protocols for transmission electron microscopy were carried out as described by Lee and Taylor [17].

### 3.3. PCR Amplification and Cloning of 16S rRNA Gene

High quality DNA was isolated using a GenEluteTM Bacterial Genomic DNA Extraction Kit according to the manual procedures (Sigma-Aldrich, USA). PCR amplification was performed in a 25 μL reaction using thermostable DyNAzymeTM EXT DNA polymerase (Finnizymes, Finland) in a PTC-200 thermal cycler (MJ Research, USA). The reaction mixture consisted of 1 × PCR buffer; 2.0 mM of MgCl2, 0.2 mM of dNTPs, 2 μM of forward and reverse primers, 100 ng of bacterial genomic DNA as template and 2.5 U of the enzyme mix. The 16S rRNA gene was amplified with the universal F8 (5′-AGAGTTTGATCMTGGCTC-3′) and rP2 (5′-ACGGCTACCTTGTTACGACTT-3′) primer pair [18]. Amplification reaction was carried out with the following cycling conditions: primary denaturation for 3 min at 95 °C, followed by 30 cycles of 30 s at 94 °C, 1 min at 55 °C and 2 min of 72 °C, and a final extension of 10 min at 72 °C. The PCR product was run through a 1% agarose gel and purified using QIA Quick Gel Extraction Kit (Qiagen, Valencia, CA, U.S.). The TOPO TA Cloning^®^ Kit (Invitrogen, U.S.) was used for cloning of the PCR product following the manufacturer’s protocol. Minipreparation of plasmid DNA was carried out using the QIAprep Spin miniprep kit (Qiagen, Valencia, CA, U.S.), followed by restriction endonuclease analysis to identify the clones for sequencing. DNA sequencing was performed using an ABI Prism Dye Terminator Cycle Sequencing Ready Reaction kit and ABI PRISM 3100 Genetic Analyzer (Perkin-Elmer, Foster City, CA) following the manufacturer’s instructions. The 16S rRNA gene sequences were aligned using ClustalW and analyzed by neighbor-joining (NJ) using the MEGA 4 program [19].

## 4. Conclusions

Our phenotypic observations, biochemical analysis and genetic studies lead us to conclude that the isolated strain belonged to the *E. mallotivora* species and is the main causal agent of papaya dieback disease in Peninsular Malaysia. This identification is critical not only for further understanding of the pathogenic mechanism, but concurrently for development of suitable control strategies, including selection and development of resistant varieties to overcome the disastrous effect of this disease.

## Figures and Tables

**Table 1:** Distinguishable characteristics of *E. mallotivora* and *E. papaya.*

Characteristic	*E. mallotivora* (BT-Mardi)	*E. mallotivora* [7]	*E. papaya* [5]
Blue pigment on King’s B agar	−	−	+
Citrate utilization	+	+	−a
Reducing substances from sucrose	+	+	−
d-Mannitol	+	+	−
l-Arabinose	−	−	+

a More than 70% of the strains negative.
